# Supplementary material for: Factors that determine women’s autonomy to make decisions about sexual and reproductive health and rights in Nepal: A cross-sectional study
Source: PLOS Glob Public Health. 2023 Jan 26;3(1):e0000832. doi: 10.1371/journal.pgph.0000832 (PMC10022137; doi:10.1371/journal.pgph.0000832)
Supplement: Attachment — Submitted filename: Response Letter.docx [file pgph.0000832.s001.docx]

**Rebuttal Letter**

Date: 26^th^ June 2022

To,

The editorial manager and respected reviewers

PLOS One

Global Public Health

Dear Sir/Madam

Firstly, we all authors would like to thank the reviewers for their valuable feedback and further guidance on submission number **PGPH-D-22-00866**. We would also like to thank the editorial team of PLOS One/Global Public Health journal for considering the revision of the manuscript. We appreciate the time and effort put forth by the reviewers and editors which have greatly helped us to improve the quality of our manuscript.

Acknowledging the valuable remarks made by reviewers and editors, we have revised our manuscript accordingly. The changes and revisions are highlighted in yellow colour in the revised manuscript. If any responses are unclear or you wish additional changes, please let us know.

The responses for feedback are as follow:

**Revisions and updates on journal requirements:**

1. Please amend your detailed Financial Disclosure statement. This is published with the article, therefore should be completed in full sentences, and contain the exact wording you wish to be published.

**Response:** We have revised the **financial disclosure** statement in the revised manuscript as per guidance, **line no. 317, Page no: 21**.

2. Please update the 'Competing Interests' statement with this "The authors have declared that no competing interests exist".

**Response:** We have updated the **Competing Interests** statement in **line no. 315, Page no: 21.** as per guidance.

3. We have noticed that you have uploaded Supporting Information files, but you have not included a list of legends. Please add a full list of legends for your Supporting Information files after the references list.

**Response:** We have updated the supporting information section in **line no. 307-313, Page no: 21**. as per the reviewer’s guidance and follow the **guideline given on the PLOS One website** on the **Captions section** (<https://journals.plos.org/plosone/s/supporting-information>).

4. Please review your reference list to ensure that it is complete and correct. If you have cited papers that have been retracted, please include the rationale for doing so in the manuscript text, or remove these references and replace them with relevant current references. Any changes to the reference list should be mentioned in the rebuttal letter that accompanies your revised manuscript. If you need to cite a retracted article, indicate the article’s retracted status in the References list and also include a citation and full reference for the retraction notice.
**Response:** We have revised the reference list adhering to the guideline of the manuscript. We have rearranged the reference list as there was a duplication of some literature in the initial manuscript. We used the Mendeley reference manager tool and follow the instructions given in PLOS One submission guidelines (<https://journals.plos.org/plosone/s/submission-guidelines#:~:text=In%20the%20text%2C%20cite%20the,not%20include%20citations%20in%20abstracts>.) and instruction given in formatting reference section: <https://www.nlm.nih.gov/bsd/uniform_requirements.html>

Similarly, we have kept recommended citations for some government, organizational and official documents and they are references no: **13, 14, 15, 16, 23, 35, 42 and 46.**

Finally, our English language review had suggested amending the **title,** so we now named our title “Factors that Determine Women’s Autonomy to Make Decisions About Sexual and Reproductive Health and Rights in Nepal: A Cross-Sectional Study**”** instead of “Factors that Determine Women’s Autonomy in Decision Making for their Sexual and Reproductive Health and Rights in Nepal: A Cross-Sectional Study”.

**Reviewer #1:** Overall the secondary analysis was conducted correctly with interesting findings. However some of findings can be a basis for the further studies. For instance on p.14 ratio of autonomous Muslims is twice less than ratio of autonomous Janajati. The line 159 and 160 states on absence of any association of autonomy with religion. Interesting finding is that higher education of women has more positive impact on autonomy than high education of their husbands. Could be interesting to see the cases where both are highly educated. Exposure to the media is associated with autonomy but it could be one of the possible factors among few which influences these women autonomy. Perhaps can be other factors along with exposure to media which leads to the greater autonomy. Impact form exposure to media depends on the context which media has and in Nepal mass media probably does talk about women rights. Unfortunately its not the case in all countries to the same extend and authors can be cautious with replication of these recommendation to other country contexts.

**Response:** Thank you for pointing this out. We agree with this. We should have made it more clear in the discussion point. Since, the Government of Nepal has categorized Muslims as enthic group in our country, we have also analyzed the data accordingly. However, it is also a fact that Muslim is a religion too. Therefore, we have added an explanation and recommendation as per your feedback in the **“Discussion” section, line no: 247-253, Page no 18 to make careful recommendation.**

Also agree that in Nepal mass media is means of disseminating health-related message. There are no restrictions on broadcasting sexual and reproductive health-related messages which may not be the case in other countries. Thank you so much for the valuable remarks, we have considered this suggestion in the **“Discussion” section, line no: 207-214, Page no 16- 17.**We have also considered the recommendation for further study to explore women’s autonomy when both partner have higher education in the **“Discussion” section, line no: 222-226, Page no:17.**

**Reviewer #2:** The study entitled “Factors Determining Women’s Autonomy in Decision Making for their Sexual and Reproductive Health and Rights” by Adweeti et al. discusses the factors that contribute to women’s autonomy in decision making on their sexual and reproductive health and rights (SRHR). The authors have written on a very important and interesting topic. However, some points should be considered in the revision of the manuscript:

1. There are some typographical and grammatic errors within the text that require correction to improve legibility.

**Response:** Thank you so much for pointing this out. We realized there were so many typos and grammatical errors. As per your feedback, we consulted with the **English lecturer and the entire manuscript has undergone substantial English editing**.

2. The introduction outlines the progress and commitments made by Nepal in improving women’s decision-making in their sexual and reproductive health and rights. The authors should consider adding additional literature on how their study relates to previously published research on women’s decision-making on their SRHR.

**Response:** We appreciate the valuable remarks. We have searched for research conducted in Nepal on the women’s decision-making on sexual and reproductive health and found studies like **references no. 8 and 20** which have tried to explore the women’s empowerment and its association with in making sexual and reproductive health decisions. We have tried to **relate our study with those previous studies in the “Introduction” section, line no: 75-79, Page no: 4.**

3. The authors should consider checking the measurement of the dependent variable and the cited reference [15] which does not have the questions used to measure autonomy.

**Response:** Thank you for the critical feedback. The Nepal Demographic and Health Survey 2016 has considered sustainable development goal 5 (gender equality), target 5.6 “5.6.1 Proportion of women aged 15-49 years who make their own informed decisions regarding sexual relations, contraceptive use, and reproductive health care” to look after women’s empowerment. Thus, we have adapted the definition to consider women’s autonomy in exercising their sexual and reproductive health rights and taken as a dependent variable for this analysis. We have explained this further in the **“Definition of Variables” section, Table 2, line no: 118, Page no: 6-7.**

Once again, we thank all the reviewers and the editorial team for your valuable contributions. We have tried to address every feedback at our best. We hope that the revisions in the manuscript and our accompanying responses will be satisfactory to make our manuscript suitable for publication in the PLOS One Global Public Health journal.

Sincerely,

Adweeti Nepal,

Santa Kumar Dangol,

Sujan Karki and

Niraj Shrestha
